# Supplementary figures and images for: Epidemic management and control through risk-dependent individual contact interventions
Source: PLoS Comput Biol. 2022 Jun 23;18(6):e1010171. doi: 10.1371/journal.pcbi.1010171 (PMC9223336; doi:10.1371/journal.pcbi.1010171)

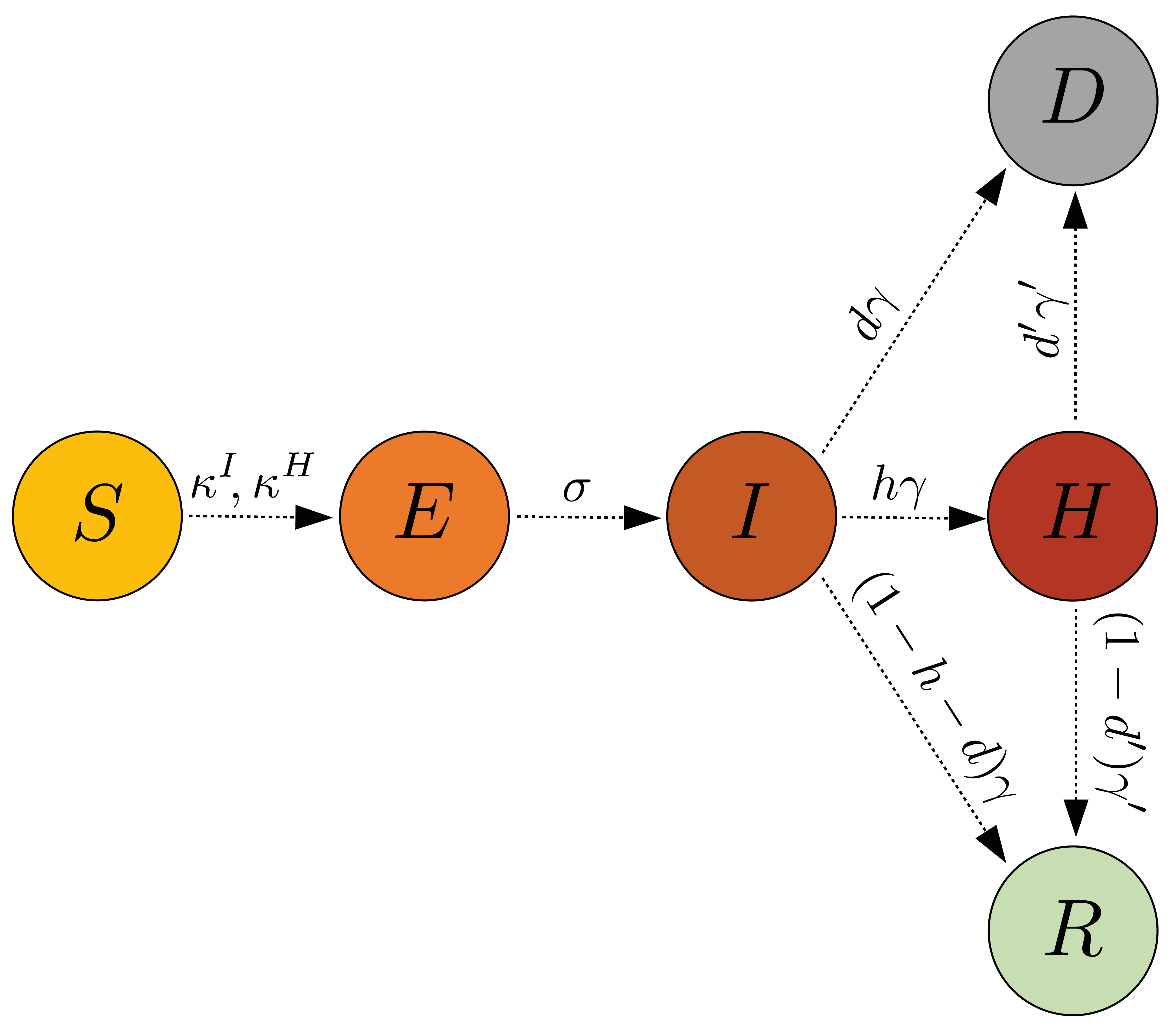

Supplement: S1 Fig — Infected and hospitalized nodes infect susceptible nodes at rates κI and κH, respectively. After being infected, susceptible nodes become exposed. Exposed nodes become infectious at rate σ. Infected nodes may get hospitalized at rate hγ, die at rate dγ, or become resistant at rate (1 − h − d)γ. Once hospitalized, nodes either become resistant at rate (1 − d′)γ′ or die at rate d′γ′. (TIF) [file pcbi.1010171.s001.tif]

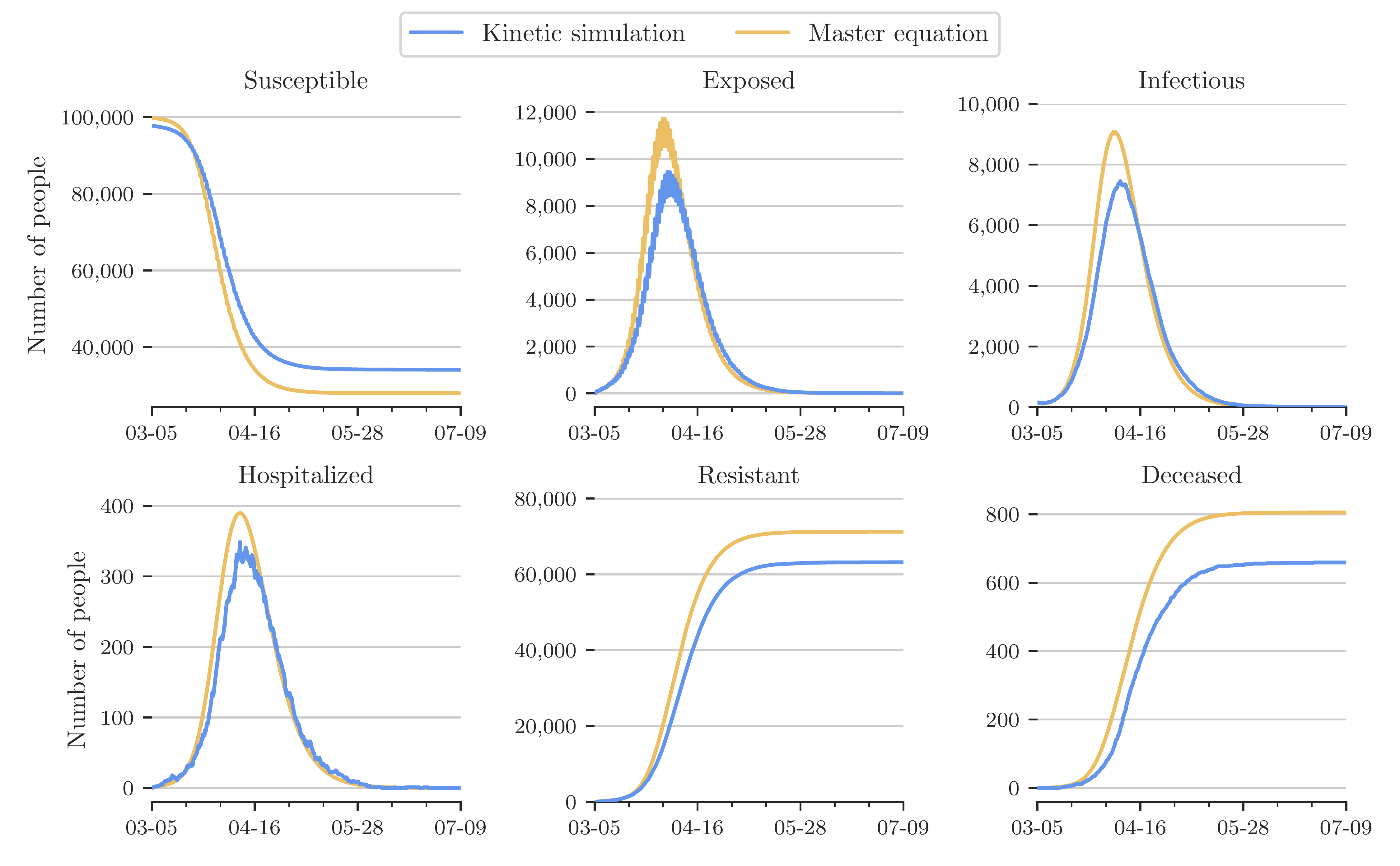

Supplement: S2 Fig — (TIF) [file pcbi.1010171.s002.tif]

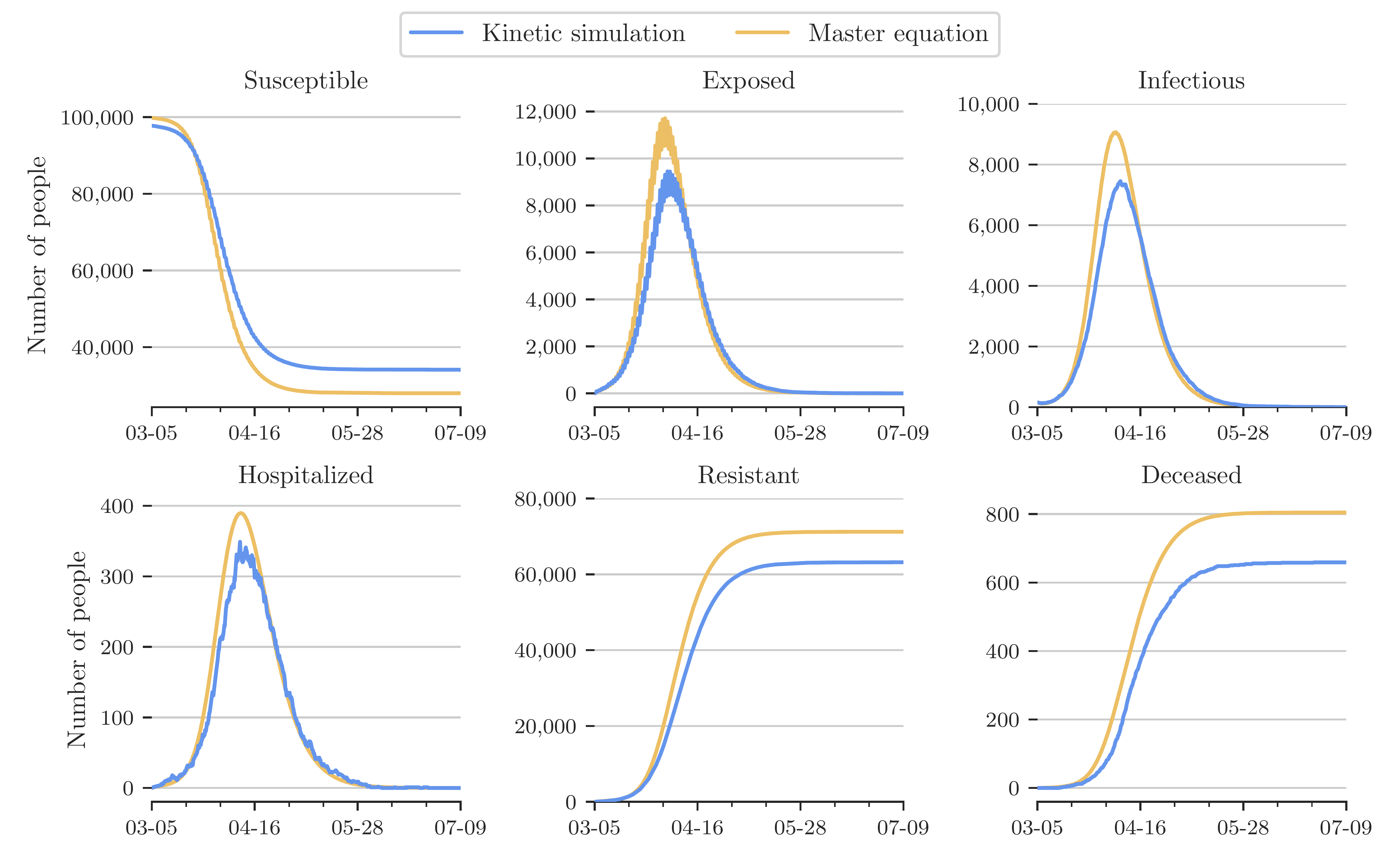

Supplement: S3 Fig — (TIF) [file pcbi.1010171.s003.tif]

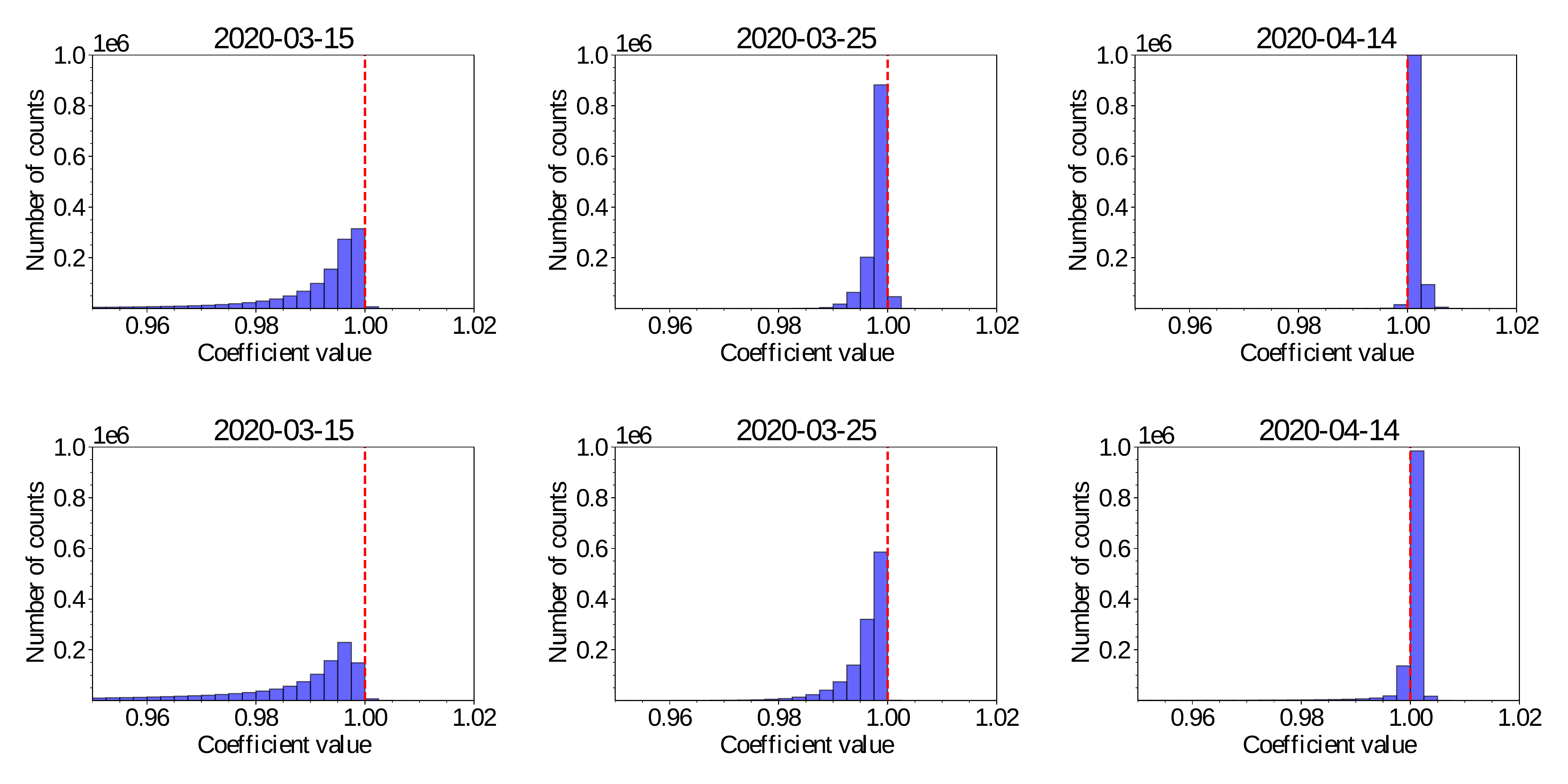

Supplement: S4 Fig — (TIF) [file pcbi.1010171.s004.tif]

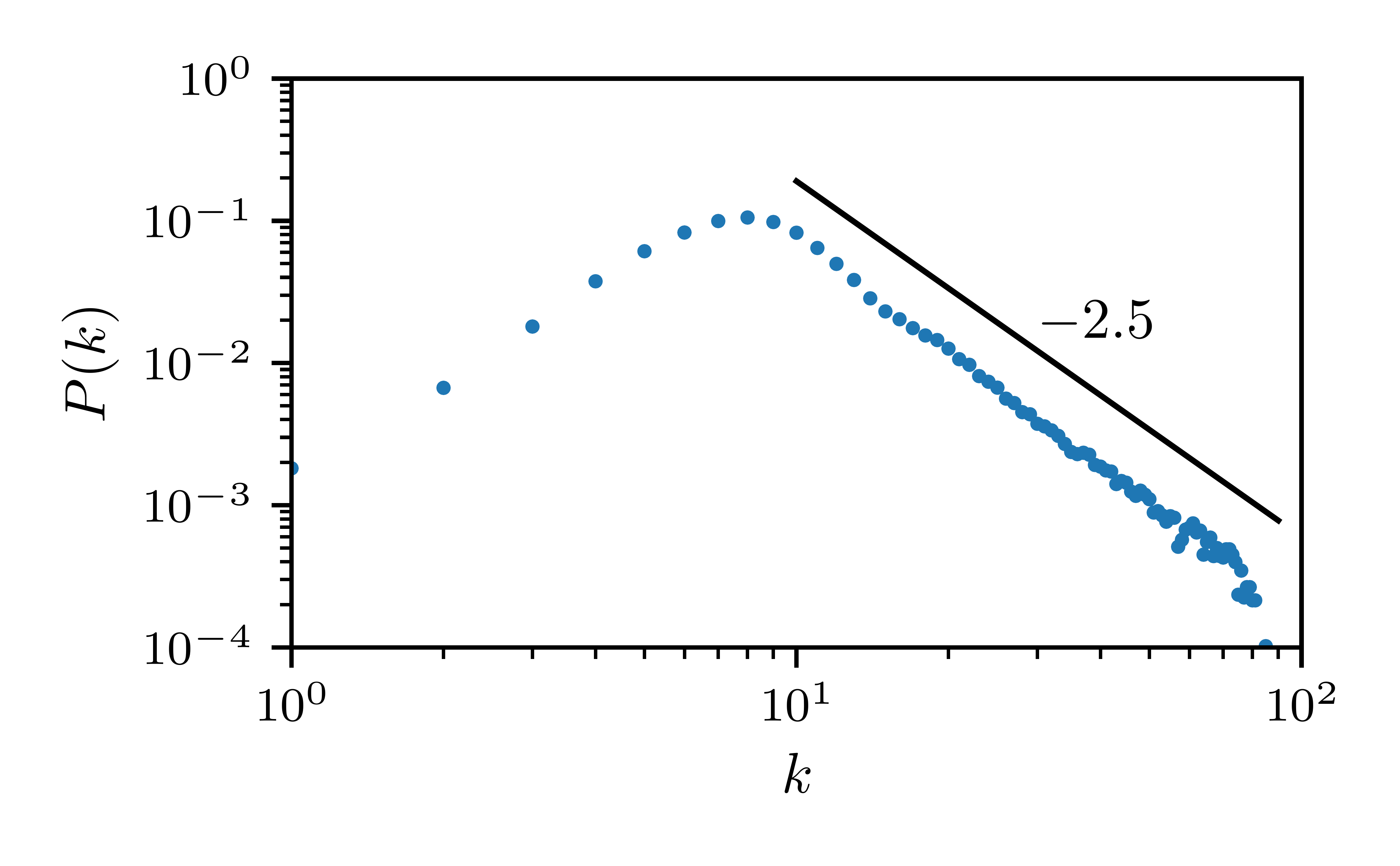

Supplement: S5 Fig — (TIF) [file pcbi.1010171.s005.tif]

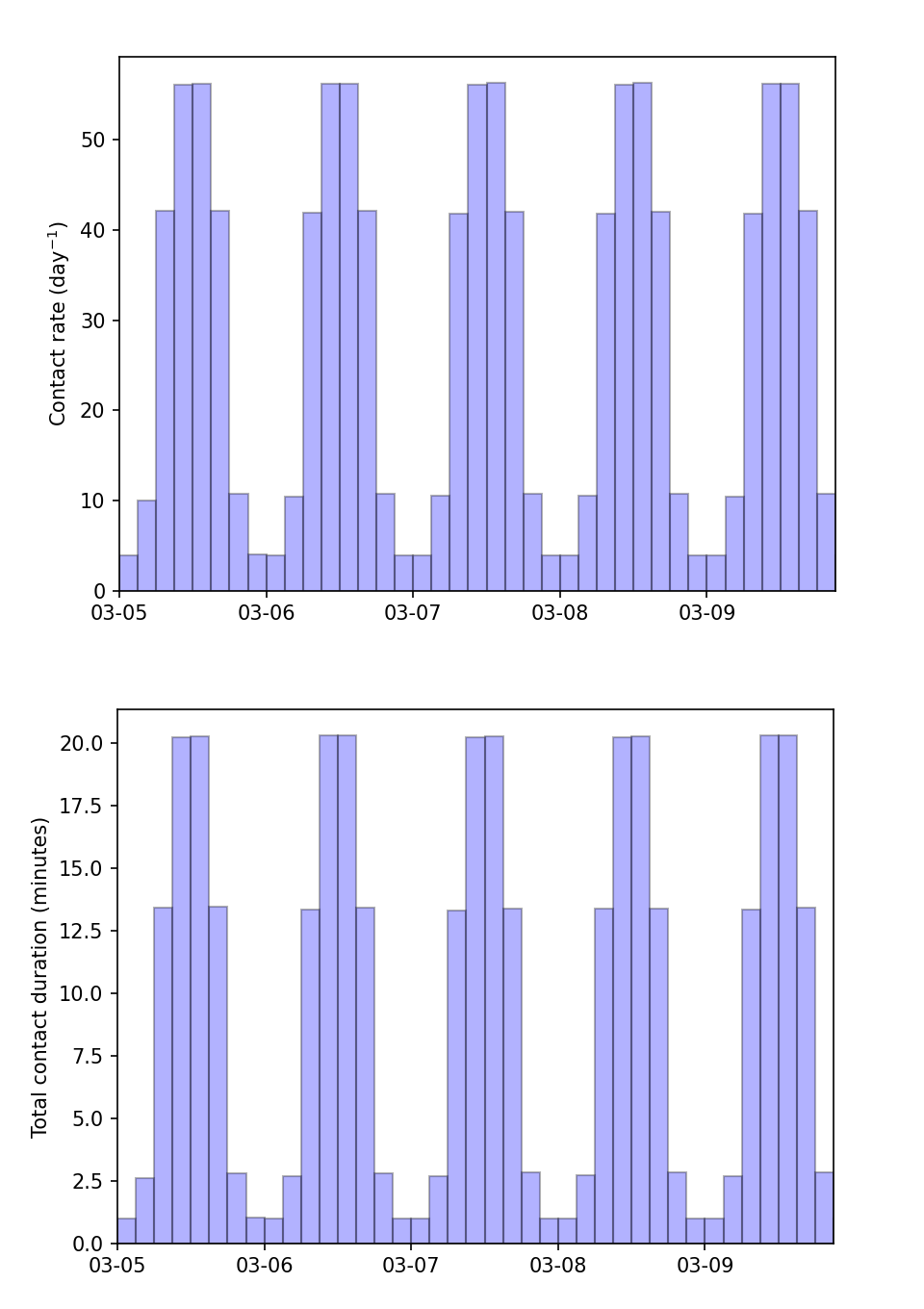

Supplement: S6 Fig — Displayed are the ensemble-averaged and node-averaged contact rate and total contact duration. (TIF) [file pcbi.1010171.s006.tif]

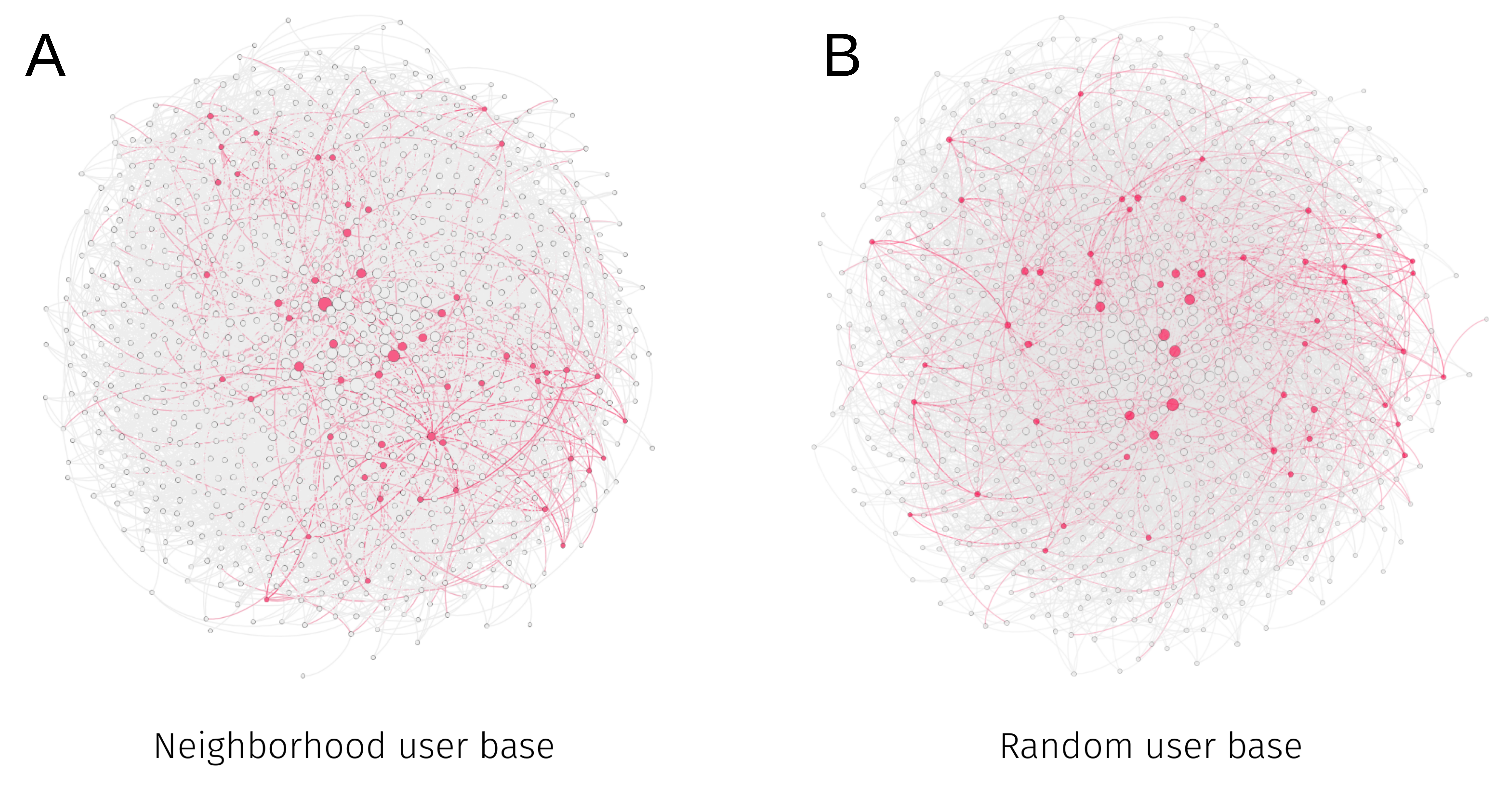

Supplement: S7 Fig — (a) Neighbor-based user base, constructed by iteratively adding neighborhoods. (b) Random subnetwork of users. Red nodes and edges are part of a user base, grey nodes and edges of the overall population. The shown networks have 982 nodes and 5,916 edges. Both user bases contain 5% of all nodes. (TIF) [file pcbi.1010171.s007.tif]

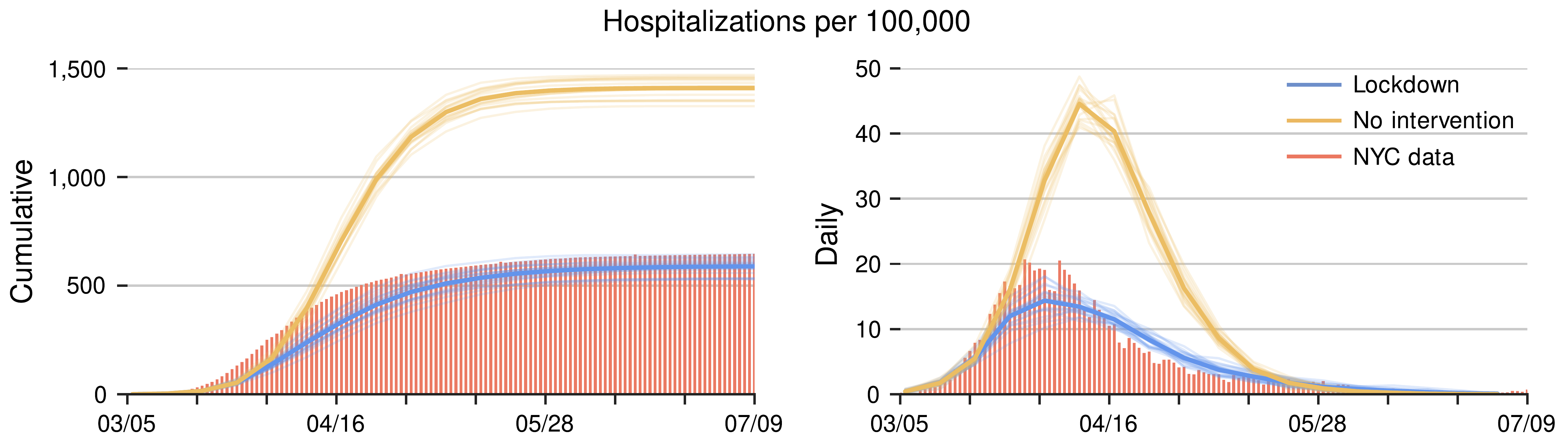

Supplement: S8 Fig — The left panel shows cumulative hospitalizations and the right panel daily hospitalizations per 100,000 population for the same simulations as those in Fig 2. Red bars represent COVID-19-related hospitalization rates for New York City [36]. As in Fig 2, the simulation data are smoothed with a 7-day moving average filter. (TIF) [file pcbi.1010171.s008.tif]

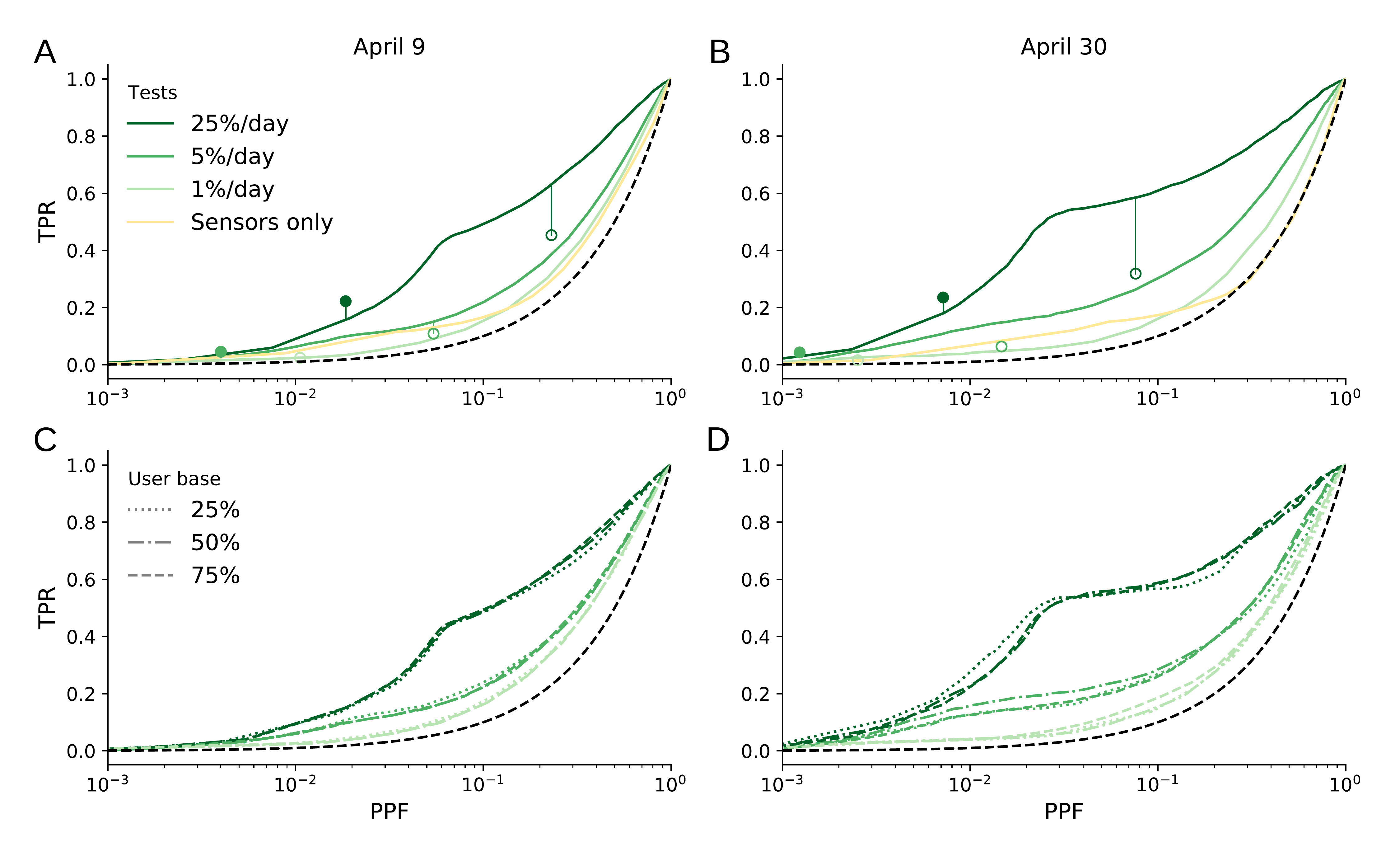

Supplement: S9 Fig — As in Fig 3, but for subnetworks with randomly selected nodes rather than for subnetworks with a neighborhood topology. For the filled circles, the 1%/day case falls outside the plotting region; values for panel (A) are (7×10^−4^, 0.009) and for panel (B) are (2×10^−4^, 0.01). (TIF) [file pcbi.1010171.s009.tif]

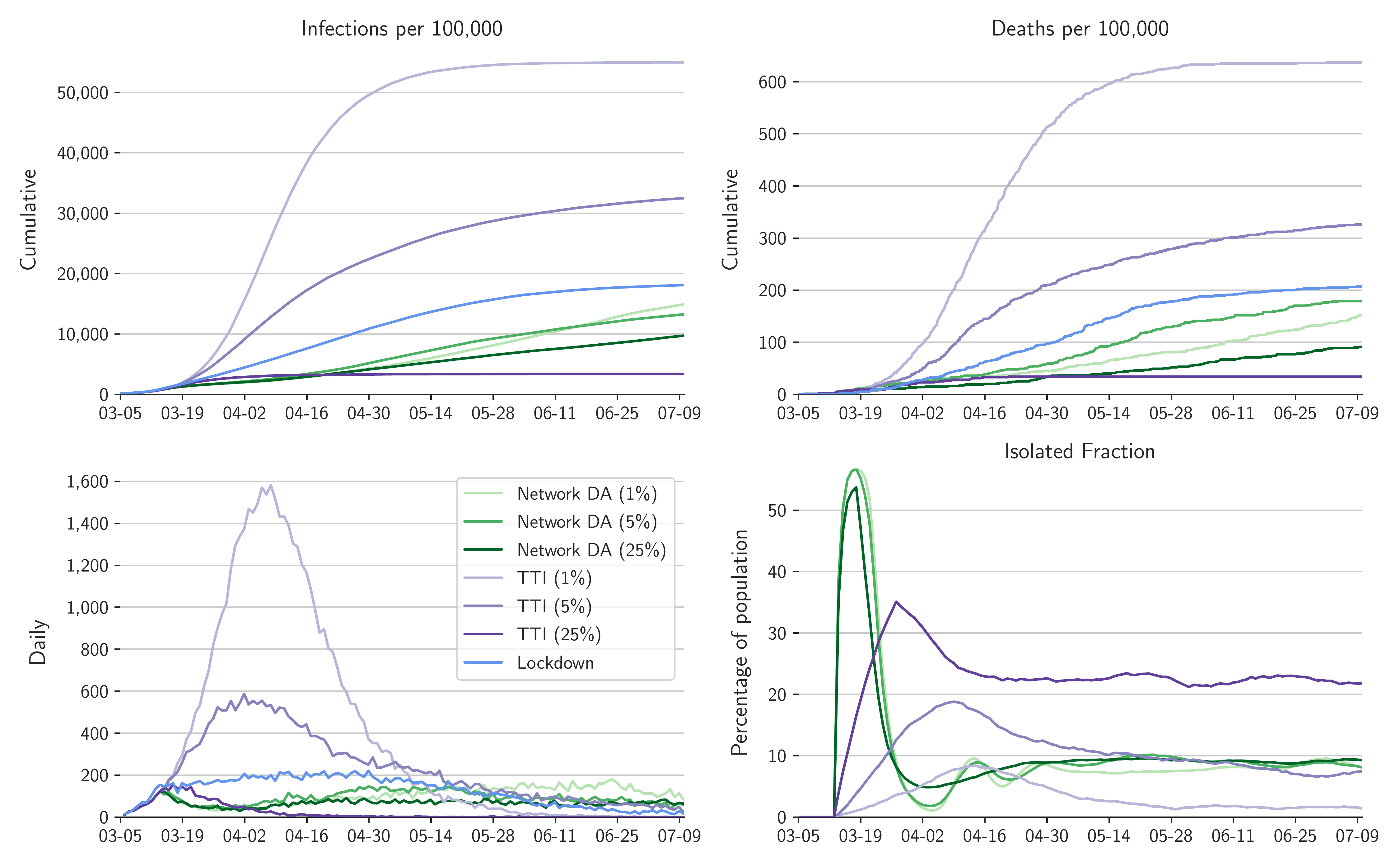

Supplement: S10 Fig — As in Fig 5, but with a subnetwork with randomly selected nodes and with a classification threshold cI = 0.25%. (TIF) [file pcbi.1010171.s010.tif]

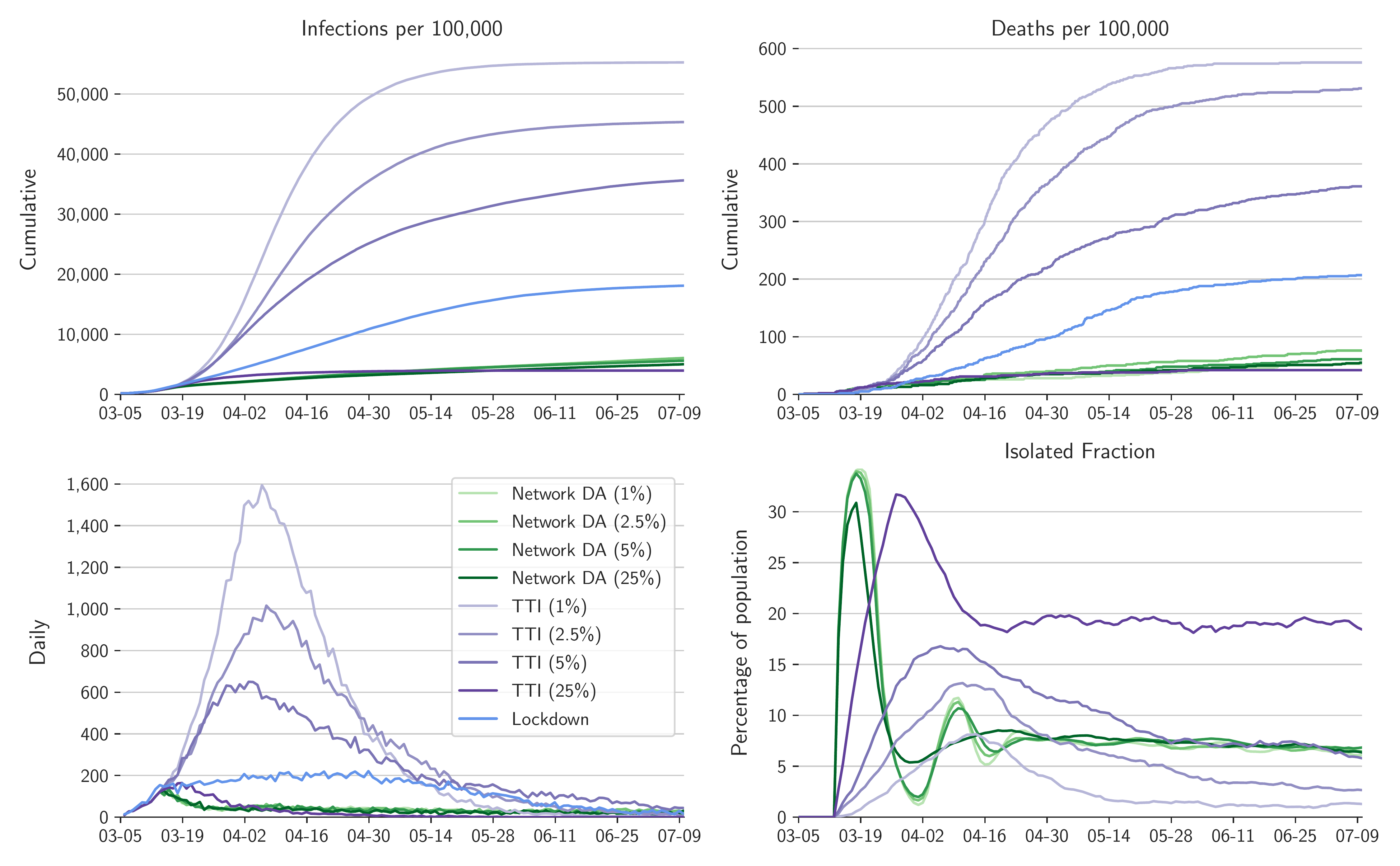

Supplement: S11 Fig — (TIF) [file pcbi.1010171.s011.tif]

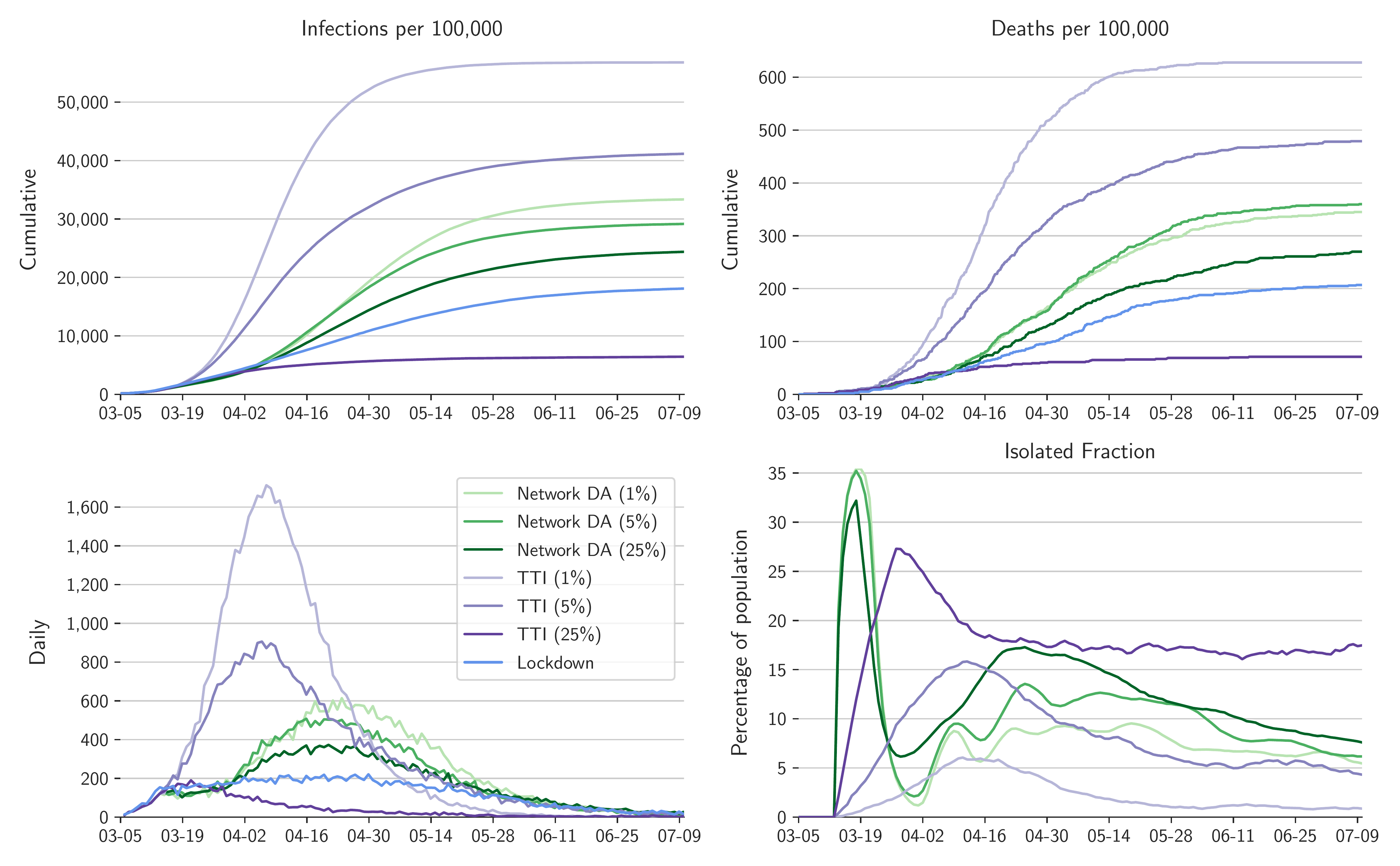

Supplement: S12 Fig — As in S11 Fig, but with a subnetwork with randomly selected nodes. (TIF) [file pcbi.1010171.s012.tif]

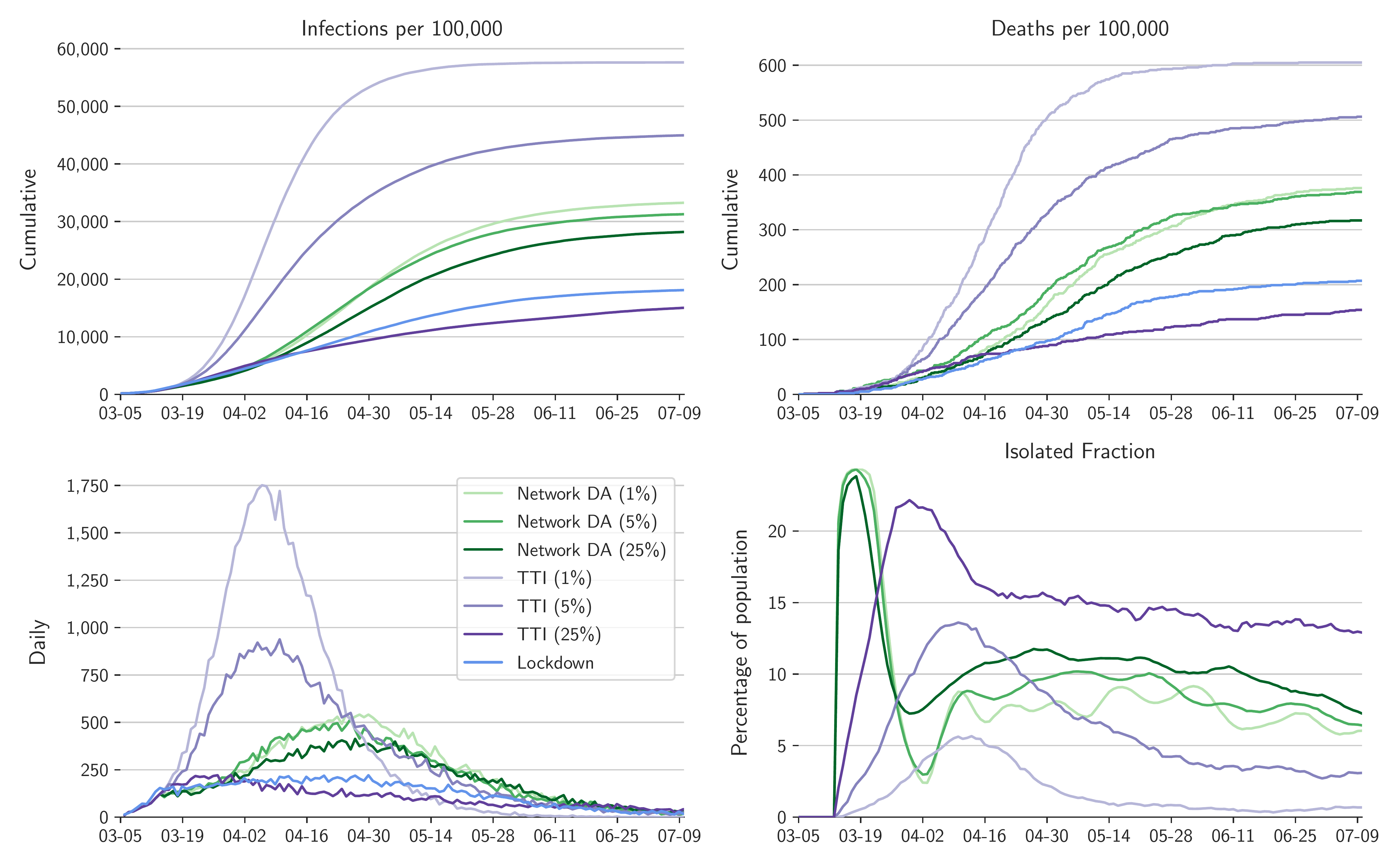

Supplement: S13 Fig — (TIF) [file pcbi.1010171.s013.tif]

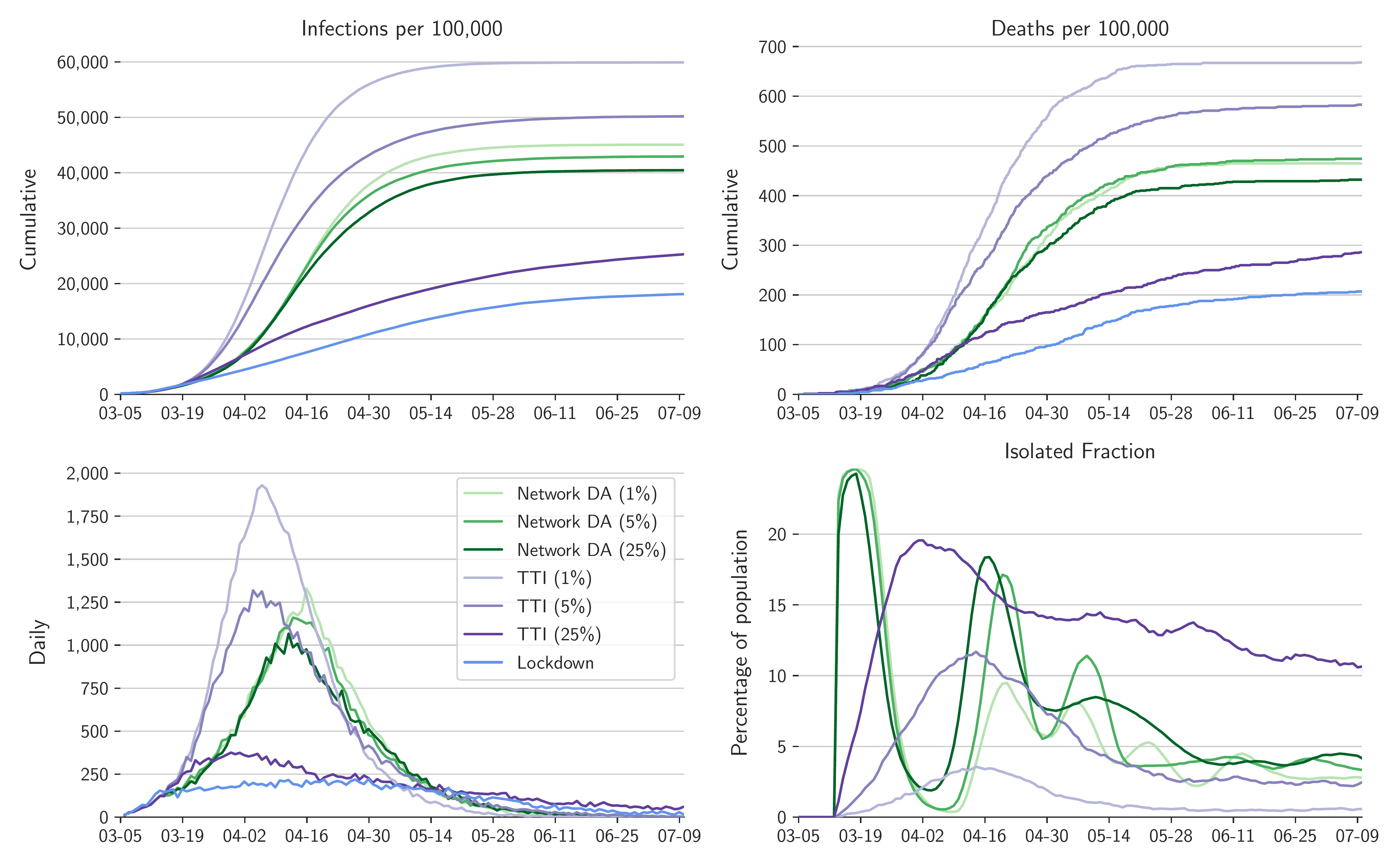

Supplement: S14 Fig — As in S13 Fig, but with a subnetwork with randomly selected nodes. (TIF) [file pcbi.1010171.s014.tif]

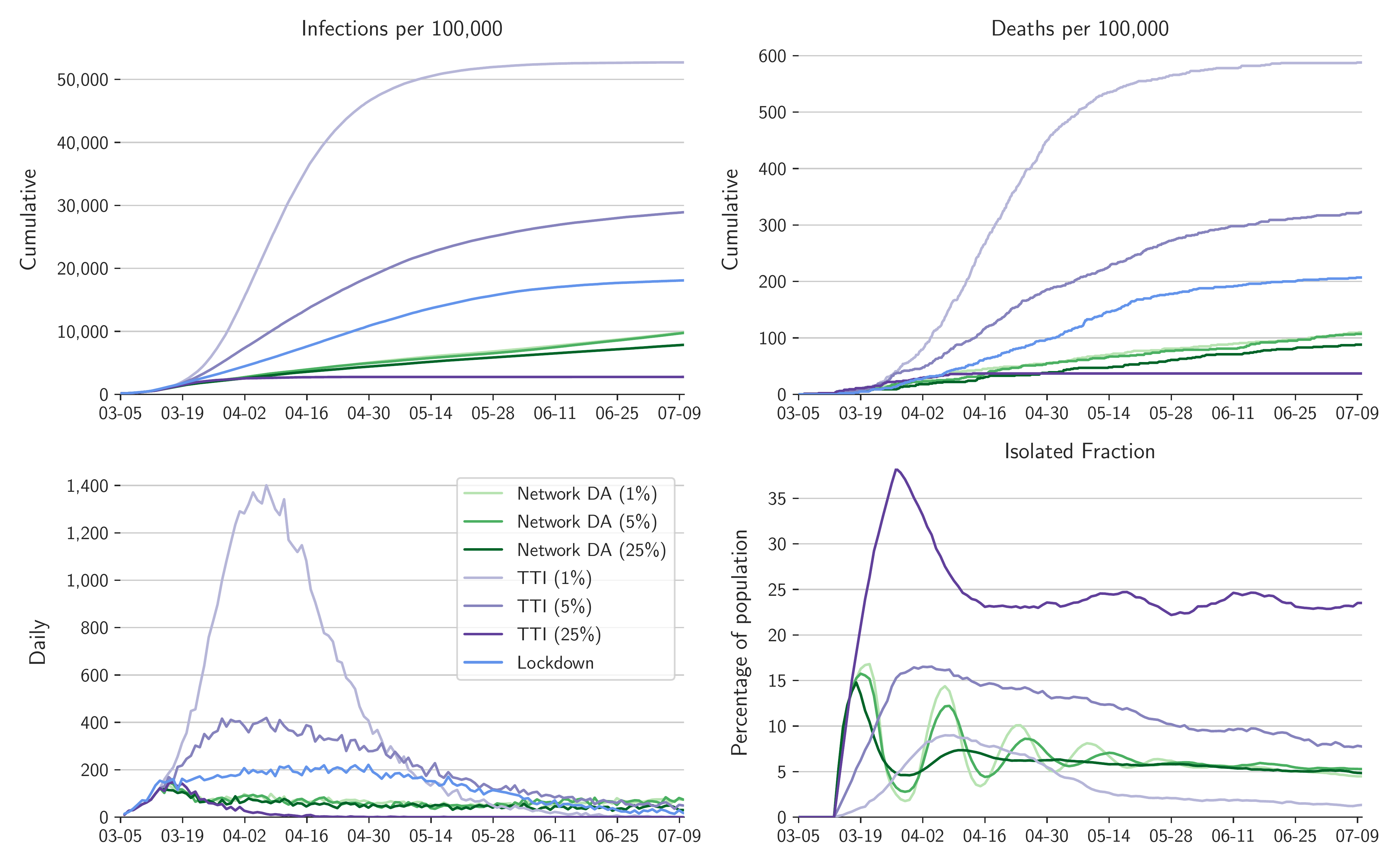

Supplement: S15 Fig — (TIF) [file pcbi.1010171.s015.tif]

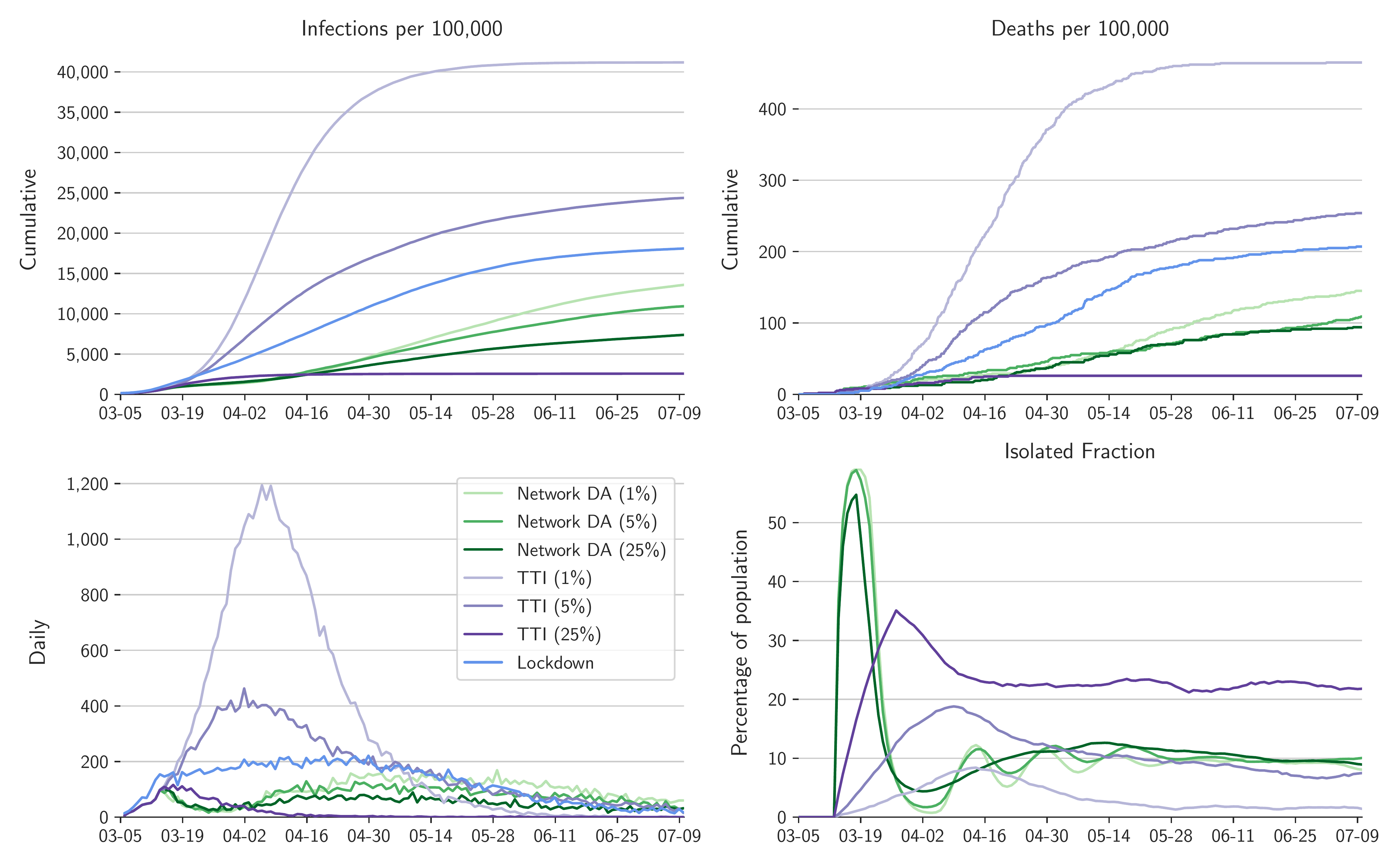

Supplement: S16 Fig — (TIF) [file pcbi.1010171.s016.tif]

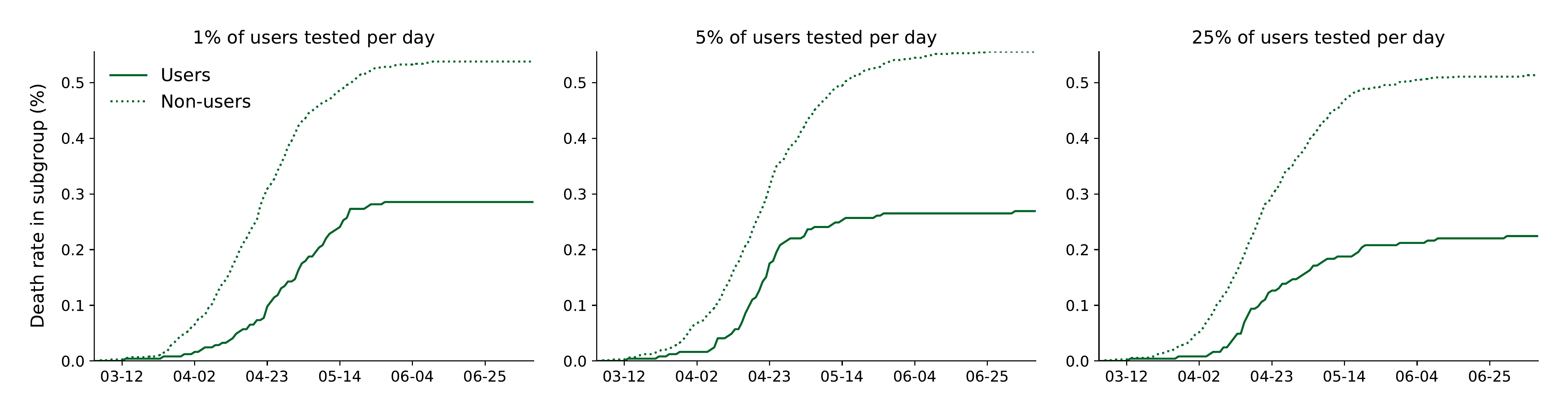

Supplement: S17 Fig — Individual contact interventions are applied within the user base from March 15 onward. (TIF) [file pcbi.1010171.s017.tif]

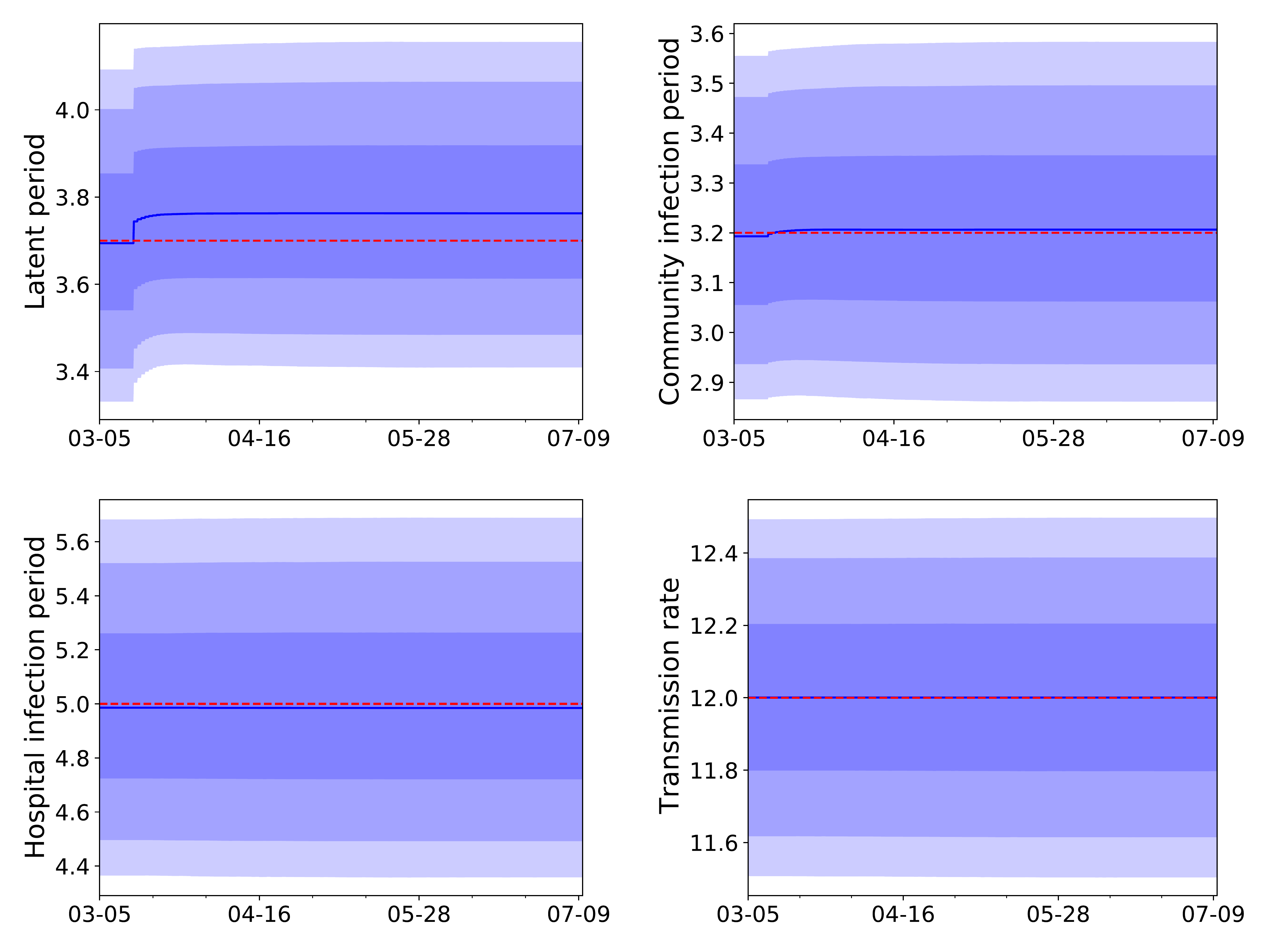

Supplement: S18 Fig — The shaded regions contain 50%, 80% and 90% of the distribution. The dashed line represents the true parameters in the stochastic simulation. During the first 8 days, no DA is performed, and the parameter distributions are the prior distributions. (TIF) [file pcbi.1010171.s018.tif]
